# Enhancing Muscle Strength Post Anterior Cruciate Ligament Reconstruction: A Case Report Assessing the Effectiveness of Russian Current

**DOI:** 10.7759/cureus.54593

**Published:** 2024-02-21

**Authors:** Khushi M Gandhi, Grisha Ratnani, Nishigandha P Deodhe, Krishnayani Shende

**Affiliations:** 1 Neurophysiotherapy, Ravi Nair Physiotherapy College, Datta Meghe Institute of Higher Education and Research, Wardha, IND

**Keywords:** acl tear, physical therapy rehabilitation, strengthening exercises, russian current, medial meniscus tear

## Abstract

The effectiveness of Russian electrical stimulation in enhancing muscular strength after anterior cruciate ligament (ACL) restoration is examined in this case study. In addition to traditional physiotherapy, a 29-year-old male athlete having ACL repair took part in a Russian contemporary rehabilitation regimen. Subjective evaluations of pain and functional tests all showed a substantial increase in muscular strength following the intervention. The Russian current's distinct waveform and high-frequency bursts appeared to improve neuromuscular control and quicken the activation of skeletal muscle in the excitation-contraction phase. The evaluation conducted after the intervention revealed notable enhancements in muscular strength and scores on the lower extremity functional scale. The initial score of 32, indicating a moderate functional limitation, improved to 64, indicating a minimal functional limitation. Additionally, the use of Russian electrical stimulation in ACL rehabilitation programs resulted in a reduction in pain levels from 9/10 to 3/10, as measured by the visual analog scale. These findings suggest that the implementation of Russian electrical stimulation shows promise in ACL rehabilitation. However, to validate and further explore these results, it is necessary to conduct larger-scale research studies and randomized controlled trials.

## Introduction

The knee joint is created by the femur, tibia, and patella, forming a hinge joint that is surrounded by a joint capsule containing synovial fluid. Key muscles involved in knee movement include the quadriceps femoris for extending the knee, hamstrings for flexion, and gastrocnemius and soleus for additional support. Important ligaments, such as the anterior cruciate ligament (ACL) and posterior cruciate ligament (PCL), prevent excessive forward and backward movement, while the medial and lateral collateral ligaments provide stability against medial and lateral forces. Together, these structures allow the knee to maintain a delicate balance between mobility and stability, enabling a wide range of movements necessary for everyday activities. The knee joint further depends on all its ligaments and menisci for static as well as dynamic stability because its articular surfaces are incongruent [1]. These structures include the menisci, the capsule, and the muscles that cross the joint [2]. The menisci are two semilunar fibrocartilage structures with a cross-sectional shape resembling a wedge that is connected to the patella, femur, and tibia. The lateral meniscus appears in an O shape, whereas the medial meniscus has a C shape. Injuries to the ACL are the most frequent ligament damage so far [3]. These wounds have the potential to end an athlete's profession and leave everyone else seriously disabled [4]. It is a very common injury, mostly to athletes, as they have to use their knee for the majority of actions, such as jumping and kicking. Due to its confrontation with anterior tibial conversion and rotating bearing, the ACL is a vital structure of the knee joint [5]. The ACL is 32 mm in mean length and 7-12 mm in width when the knee is kept in extended position. The posterolateral bundle (PLB) and the anteromedial bundle (AMB) are the two major components of the ACL [6]. The primary difference between them is that with flexion, AMB lengthens, and PLB shortens in size [7]. A net of all the proteins, including glycoproteins elastic systems and glycosaminoglycans, with numerous useful interactions, comprise the matrix of the ACL, which is microstructural with collagen bundles of several types (mostly type I). The ACL can tolerate a variety of tensile strains and multiaxial stresses because of its high elastic system and complicated ultrastructural arrangement [8]. The posterior articular branch of the tibial nerve innervates the ACL, while divisions of the genicular artery supply blood to it.

Utilizing this population-based data, thorough descriptive epidemiology can pinpoint intervention programs aimed at lowering these injuries, find high-risk subdivisions within the overall inhabitants, and shed light on the circumstances and processes of injury as well as a choice of management, whether ACL reconstruction or meniscectomy is required [9,10]. Here, the ACL tear is associated with another knee menisci injury, which is also a criticism for a sportsperson. Meniscal injuries are frequently occurring injuries sustained while participating in sports, especially by football and ski athletes. Meniscal tears are commonly accompanied by pain, swelling, and functional limitation [11]. Epidemiological studies revealed that in various sports athletes, meniscal lesions include 24% injury to the medial meniscus, 8% to the lateral meniscus, and roughly 20-30% of meniscal lesions are linked to other ligament injuries [12]. To cope with ACL tears, surgical, pharmacological, and even physical therapy is advantageous. Here, we will study how Russian current (RC) could be beneficial for the following case.

Physical therapy makes extensive usage of electrical stimulus, and RCs have been promoted as a means of boosting muscular strength [13]. Intermittently moderated at a frequency of 50 Hz with 50% of duty cycle, frequency of 2.5 kHz. It is advised to treat for 10 minutes per stimulation session, with the stimulus given for 10 seconds during the "on" phase and 50 seconds during the "off" or rest period [13]. Although many of the dues seem to be unreliable, this stimulation treatment given once a day over weeks has been said to generate force gains [14].

## Case presentation

Patient information

It is a case of a 29-year-old male resident of the Akola district who is a cricket player. He experienced right knee pain while playing a sport a few months ago, and the pain only got worse; it was dull, aching in nature, started suddenly, and progressed over time. Medication and rest have so far been able to relieve it, but going about daily tasks has made it worse. He then went to the orthopedics with complaints of right knee pain on the knee in the medial side. After undergoing further investigations, including MRI and X-ray, it was determined that the patient had an ACL tear along with a medial meniscus tear. He opted out of the recommended surgery and continued receiving medication, including tablet (tab.) Tendoheal, tab. Zerodol-SP, and tab. Pantoprazole. The patient has no history of diabetes mellitus, hypertension, or trauma. Also, the patient didn’t have any prior episodes of ACL injury. No such cases were found in the patient's family. However, from what is known, the condition worsened. Five months later, the patient had a right knee arthroscopy. Given the patient's complaints regarding limited range of motion (ROM) during activities, musculoskeletal physical therapy was recommended as an additional course of treatment.

Clinical findings

The physical examination was carried out with the patient's verbal consent. Upon observation, the patient exhibited a mesomorphic build, was supine in their position, and was well-oriented to the time, place, and person. The patient had negative findings of clubbing, icterus, edema, and pallor. At the time of the examination, he was hemodynamically stable. However, the patient is complaining of right knee pain, limited ROM, and difficulty walking. Although there was no swelling felt upon examination, the patient reported grade 1 tenderness and limited ROM in his right leg. A Visual Analog Scale (VAS) was measured to evaluate pain, which scored 9/10 for activity and 5/10 for rest. Also, ROM and manual muscle testing were performed.

Radiological investigations

**Figure 1:**
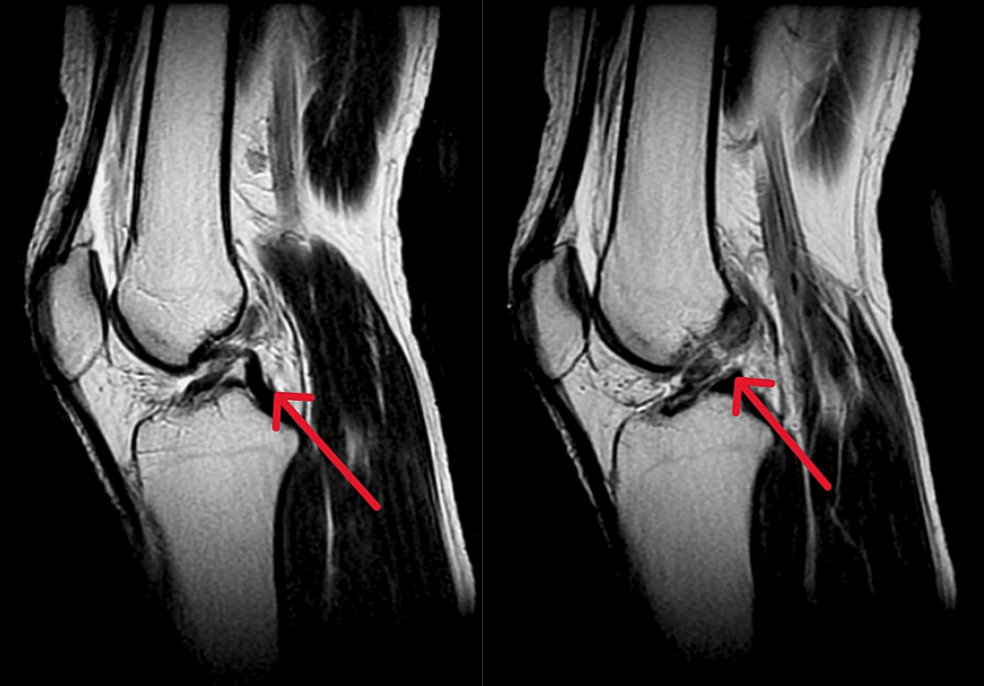
Pre-operative MRI of knee joint MRI revealing partial tear of ACL along with complex tear of medial meniscus ACL, anterior cruciate ligament

Pre-operative MRI confirmed the diagnosis of a partial tear of ACL and a complex tear of the medial meniscus, as seen in Figure 1.

Physiotherapeutic intervention

The patient was then brought to physiotherapy for additional treatment. With the consent of the patient, the treatment was started. The patient was given an RC with volitional activity along with therapeutic exercises. The treatment protocol started with the education of the patient with the condition. The patient was explained about his situation and the intervention. The main motive of the patient's rehabilitation includes muscle strengthening, improved strength, and proprioception to get ready for efficient actions, as well as increasing his ROM. In exchange, he received hamstring and gastrosoleus stretches without weight bearing, as well as patellar mobilization, heel slide exercise and wall slides, and sets of hamstrings and quadriceps training with electrical stimulation. Initially, RC stimulation was engaged on rest to avoid over-strain on muscle and to reduce pain and prevent muscle wasting. Furthermore, the implementation of hanging exercises to aid in flexion until the quadriceps muscles reach an adequate level of strength to avoid extension lag, elevating the leg in a straight position across various planes while utilizing a brace in complete extension, and performing prone leg extensions while hanging can be beneficial. These exercises were given in the first two months of the span post-surgery as phase 1 of rehabilitation.

**Figure 2:**
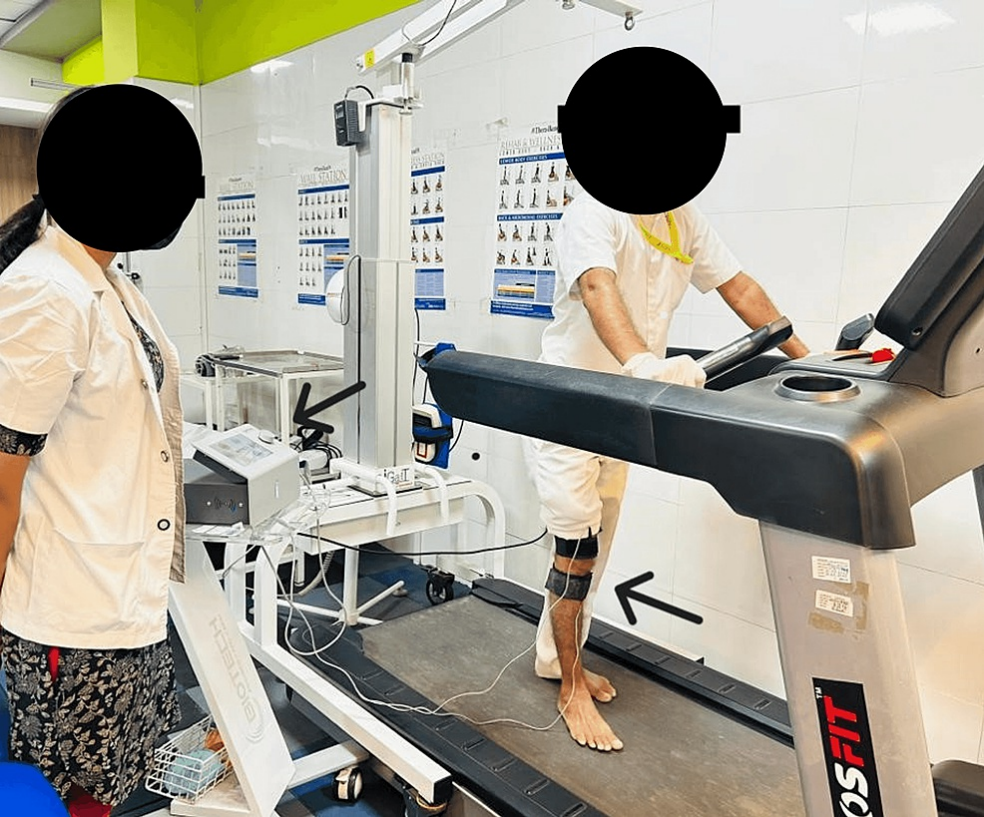
The application of Russian current on the patient with volitional activity as a treadmill

It was further associated with the RC, which was given for 10 minutes. It was given in a set of 10/50/10, where 10 repetitions were on time, and 50 reps were off time for 10 minutes. It had a duty cycle of 50%. It was given at a frequency of 2.5 kHz. The carbon rubber electrodes, with a size sufficient to encompass the quadriceps muscle, were positioned over the muscle bulk, precisely 3 inches above the patella. It was given with the volitional activity (treadmill). Figure 2 shows the patient performing exercises along with an RC.

Outcome measures

The outcome measure for the given physiotherapeutic protocol is presented in Table 1, which shows the manual muscle testing. Table 2 shows the ROM. Table 3 shows the lower extremity functional scale. Table 4 shows the VAS.

**Table 1:** Manual muscle testing by Oxford scale for right lower limb 3-: (Fair) some but not complete ROM against gravity; 3+: (Fair+) complete ROM against gravity with minimal resistance; 4: (Good) complete ROM against gravity with some (moderate) resistance; 5: (Normal) complete ROM against gravity with maximal resistance ROM, range of motion

Parameters	Pre-intervention	Post-intervention
Knee		
Flexors	3-	5
Extensors	3-	4
Hip		
Flexors	3+	5
Extensors	3+	5
Abductors	3+	5
Adductors	3+	5

**Table 2:** Range of motion for right lower limb pre- and post-intervention

Parameters	Pre-intervention (in degrees)	Post-intervention (in degrees)
Knee flexion	0°-30°	0°-80°
Knee extension	30°-0°	75°-0°
Hip abductors	0°-35°	0°-55°
Hip adduction	0°-20°	0°-42°
Hip flexion	0°-30°	0°-67°
Hip extension	30°-0°	55°-0°

**Table 3:** Lower Extremity Functional Scale (LEFS) LEFS was carried out on the patient. It is a questionnaire to check the ability of a person to do daily tasks.

LEFS	Pre-intervention	Post-intervention
Score	34, with moderate function limitation	62, with minimal function limitation

**Table 4:** Visual Analog Scale measured for pain assessment

Visual Analog Scale	Pre-intervention	Post-intervention
On activity	9/10	3/10
On rest	5/10	2/10

## Discussion

A common injury that occurs in athletes is an ACL tear. Athletes who have a medial meniscal tear may find it dangerous to regain their strength [15]. However, RC therapy has been very helpful in helping to regain muscle power and strength [16]. RC has been beneficial for low back pain as well as improving ROM of ankle and knee joint. This is a case of a 29-year-old athlete diagnosed with an ACL tear associated with a medial meniscus injury. The patient was treated with RC, and the effect of RC on strength and mobility was observed. The primary goal of physical therapy was to restore the patient's ability to carry out his everyday activities. Russian stimulation, also known as RCs, is a form of functional electric stimulation (FES) that has been proven to enhance muscle strength and power as a component of physical therapy [17]. This medium frequency generator, which operates between 2,400 and 2,500 Hz, is frequently used in conjunction with physical therapy regimens to enhance strength in the treatment of sports injuries like ACL tears [18]. Russian stimulation has been specifically utilized to treat atherogenic inhibition of the quadriceps muscle group following ACL reconstruction [19]. Russian stimulation showed evidence of continued functional recovery [20]. The use of RC has shown incredible results in patients with knee osteoarthritis suffering from knee pain and restricted ROM to improve the strength of the quadricep and relieve pain. Recuperated muscle fibers and increased cross-sectional muscle size are most likely the primary mechanisms [21].

Heggannavar et al. conducted a study on the effect of RC on osteoarthritis of the knee and came to the conclusion that in patients with primary osteoarthritis of the knee, RC stimulation is beneficial in strengthening the quadriceps muscle and enhancing functional abilities. The assessment tools employed in this study encompassed various outcome measures. These measures included the utilization of the VAS to gauge pain levels, the baseline push-pull hand-held dynamometer to quantify muscle strength in kilograms, and the Western Ontario and McMaster Universities Arthritis Index (WOMAC) Osteoarthritis Index to evaluate functional disability. It can be administered as a supplement to the current osteoarthritis care program [22].

Abd El-Latief et al. conducted a comparative analysis to investigate the impact of RC stimulation on closed kinetic chain exercise in patients with Colles' fracture. The researchers utilized ROM measurements as an outcome measure, specifically focusing on wrist flexion-extension and radial-ulnar deviation of the injured hand. Pre- and post-treatment measurements were recorded over a period of six weeks. The ROM was assessed through three repetitions, and the mean of these three trials was recorded. The findings of the study indicated that the implementation of RC stimulation, in conjunction with a traditional exercise program, demonstrated improvements in wrist ROM for patients with Colles' fracture [23].

Janarthanan et al. conducted a study to investigate the efficacy of RC and strengthening exercise in alleviating pain, enhancing strength, and improving performance in sprinters with calf muscle strain. The researchers employed the Numerical Pain Rating Scale (NPRS) to assess pain levels, the single-leg heel raise test to measure strength, and the 100-meter sprint test to evaluate speed as outcome measures. Over a period of six months, a total of 15 patients with calf muscle strains received RC treatment. The study findings indicated that the combination of RC therapy and calf muscle strengthening exercise had a significant impact on reducing pain and enhancing both strength and performance in sprinters [24].

Prabha et al. conducted a study to examine how RC therapy affects pain, quadriceps strength, and knee function in individuals with primary knee osteoarthritis. The study involved 34 patients who were split into two groups, with 17 participants in each group. The intervention involved administering RC therapy for two weeks, with five sessions per week, resulting in a total of 10 sessions. To evaluate the outcomes, the researchers used various measurement tools, such as the NPRS to assess pain levels, a hand-held dynamometer to measure quadriceps strength, and the Lysholm knee scoring scale to evaluate functional status. Based on their findings, the authors concluded that the combination of RC therapy and supervised exercises effectively reduced pain, improved quadriceps strength, and enhanced knee function in individuals with knee osteoarthritis [25].

Rajan et al. conducted a study with the aim to investigate the immediate and long-lasting impacts of interferential therapy (IFT) and RC on the strength of the quadriceps muscle in patients recuperating from lower limb fractures. A total of 38 participants, aged between 16 and 70 years, were carefully chosen based on specific criteria and then divided into three groups. Group A (n = 14) received a combination of IFT and conventional exercises (CEs), group B (n = 13) received RC and CE, and group C (n = 11) solely underwent CE for a duration of 14 days. The patients underwent electrical treatment for six days and were evaluated prior to the intervention, on the sixth day after the intervention, and on the 14th day after the intervention. The assessment included the NPRS, ROM, and isometric muscle strength. The findings of this study indicate that RC exhibits a slight advantage over IFT and CE in terms of reducing pain and enhancing muscle strength in the short term. However, CE demonstrates the ability to sustain pain relief and muscle strength over a longer period, thus indicating its effectiveness in the long term. Nevertheless, from a statistical standpoint, neither treatment proves to be superior in reducing pain or increasing muscle strength [26].

## Conclusions

This case report illuminates the potential advantages of utilizing RC therapy during the rehabilitation phase after ACL reconstruction in a 29-year-old male cricket player. Specifically, it highlights the improvements in muscle strength and functionality that can be achieved through this therapeutic approach. The patient is advised to adhere to this treatment regimen by engaging in one session lasting 10-15 minutes daily for a period of three to four weeks until full strength and recovery are achieved. Consistent daily adherence to the treatment is crucial for optimal effectiveness until complete recovery. It is recommended that the patient continues the treatment for an additional three to four weeks following phase 2 for maximum benefit. In case the patient experiences any acute infection, skin allergy, excessive pain, discomfort, muscle fatigue, or weakness, it is advisable to discontinue the therapy. The findings emphasize the importance of targeted interventions in order to enhance postoperative outcomes. This proactive approach has the potential to refine rehabilitation strategies, optimize patient outcomes, and facilitate an early return to sports following ACL surgery while also enhancing performance in later stages. Consequently, healthcare professionals and therapists are encouraged to consider incorporating RC therapy as an adjunctive intervention in their rehabilitation protocols, pending further substantiating research.
